# Novel *Bacillus subtilis* Spore-Displayed Tyrosinase Kit for Rapid Detection of Tyrosine in Urine: Pharmaceutical Applications for the Early Diagnosis of Kidney-Related Diseases

**DOI:** 10.15171/apb.2019.040

**Published:** 2019-06-01

**Authors:** Maziyar Tayebi, Afrouzossadat Hosseini Abari, Giti Emtiazi, Byung Gee Kim, Junehyung Kim

**Affiliations:** ^1^Department of Biology, Faculty of Sciences, University of Isfahan, Isfahan, Iran.; ^2^Department of Biotechnology, Faculty of Biological Sciences and Technology, Shahid Ashrafi Esfahani University, Isfahan, Iran.; ^3^School of Chemical and Biological Engineering, Seoul National University, Gwanak-ro, Gwanak-gu, Seoul 08826, Republic of Korea.; ^4^Department of Chemical Engineering, College of Engineering, Dong-A University, Busan, Korea.

**Keywords:** Chronic kidney disease, Diabetes, Health protection program, Pharmaceutical developers, Spore-displayed tyrosinase, Tyrosine

## Abstract

***Purpose:*** Simple and cheap diagnostic kit development is one of the important aims of pharmaceutical developers and companies focused on public health improvement. The *Bacillus subtilis* spore surface-display technique is a genetic engineering method that is used to develop new-generation diagnostic kits applicable for the early detection of various types of diseases. In this study, we developed a novel simple, rapid, and inexpensive diagnostic paper-based kit to detect tyrosine in urine samples of humans and animals that is applicable for home or laboratory use.

***Methods:*** The *B. subtilis* spore-displayed tyrosinase system developed by genetic engineering methods was used to prepare a paper-based kit to detect tyrosine in urine samples of different groups of patients (i.e., patients with diabetes, diabetes with chronic kidney disease (CKD), and chronic kidney disease) for the detection of tyrosine during the acute disease phase. To confirm the sensitivity and specificity of the kit, tyrosine was also detected in urine samples using conventional liquid chromatography/mass spectroscopy.

***Results:*** Different concentrations of tyrosine (0.1–1 mM) were detected in urine samples based on visible changes of color from bright brownish-gray to dark brownish-gray within 1 hour. The kit could screen samples to distinguish the three groups of patients based on formation of a broad spectrum of colors reflecting the concentration of tyrosine.

***Conclusion:*** To the best of our knowledge, this is the first diagnostic kit with potential to rapidly diagnose various diseases related to the production of tyrosine in biological samples. This kit is not only widely applicable, including for personal use in the home, but is also appropriate as a part of standard screening tests and health protection programs in countries with limited resources.

## Introduction

Tyrosine is an aromatic amino acid with a phenolic group that is synthesized from phenylalanine. As a precursor of thyroid and adrenal hormones, tyrosine has important physiological functions, and is responsible for melanin production in humans.^1,2^ Moreover, tyrosine can be used as a disease marker, since its presence in the blood and urine increases due to disorders of tyrosine metabolism. Such excretion of an amino acid with a critical role in cellular metabolism and hormonal balance can cause numerous life-threatening disorders.^3^ Tyrosinemia is a genetic disease characterized by an increase in tyrosine levels in the cells and tissues, which causes serious health problems such as liver and kidney failure, softening and weakening of the bones, hepatocellular carcinoma, and several psychological disorders.^3^ Early diagnosis of the disease in infants is the main factor to prevent disease progression. In this regard, simple and inexpensive methods for routine screening would be valuable for pharmaceutical developers and companies. There are also several lines of evidence pointing to the presence of tyrosine accumulation in diabetes mellitus and renal failure.^4,5^ Thus, elevation of tyrosine concentrations in the plasma and urine should be continuously monitored in patients with any of the above-mentioned diseases. Toward this end, most clinical laboratories generally detect urine tyrosinase using liquid chromatography or spectroscopic methods, which can be costly and time-consuming, and require specialized equipment that is not readily available, especially in underdeveloped countries.^6,7^

As one of the common substrates for tyrosinase, tyrosine is a copper-dependent enzyme with two copper atoms exhibiting monooxygenase and dioxygenase activities.^8^ The final product of the tyrosinase reaction in the presence of its substrate tyrosine is melanin, a brown pigment. Thus, many studies have employed tyrosinase as a biosensor to detect phenolic compounds,^9,10^ which requires an appropriate support for immobilization.^11,12^ The spore-surface display technique is a genetic engineering method to express a protein or enzyme on the surface of *Bacillus subtilis* spores, and has been used in several applications such as in the development of biosensors or diagnostic kits. The spores of *B. subtilis* are safe and stable under temperature and pH changes, and can withstand the effects of ultraviolet radiation.^13,14^

Therefore, we sought to develop a more easily accessible, rapid, and cost-effective paper based-kit for tyrosine monitoring in the urine using a bacterial spore-displayed tyrosinase system. In this study, we evaluated the potential efficiency of this system for screening urine tyrosinase in different patient groups, including samples from patients with diabetes, chronic kidney disease (CKD), and those with both conditions.

## Materials and Methods

### Bacterial cultivation and spore preparation

*Bacillus megaterium* tyrosinase was displayed on the surface of *B. subtilis* spores using the genetic engineering techniques described in detail in our previous work.^15^ The cells were cultivated in 1×-modified super-rich medium to increase the mass. To collect and purify the spores, the vegetative cells were inoculated into Difco sporolation medium with 5 µM chloramphenicol, and incubated for 24 hours at 37°C with shaking at 200 rpm. This medium contained 0.8% w/v nutrient broth, 0.1% KCl, 0.025% MgSO_4_.7H_2_O, 1 mM Ca(NO_3_)_2_, 0.01 mM MnCl_2_, and 0.01 mM FeSO_4_ in 1 L of distilled water, pH 7. The spores were then purified using a renografin (sodium diatrizoate, S-4506, Sigma) gradient method.

### Tyrosine monitoring in urine samples and preparation of the paper-based biosensor

The spore suspension was centrifuged for 10 minutes at 13 000 rpm, and then 60 μL of CuSO_4_ (1 mM) was added to enhance the enzymatic activity, followed by incubation at 37°C for 1 hour. The samples with different concentrations of L-tyrosine (ranging from 0.1 nM to 1 mM) were added to the mixtures (final reaction volume of 1 mL). The reactions were analyzed after 1 hour at 475 nm (ε = 3600 M-1 cm-1) on a UV-160 spectrophotometer (Shimadzu, Japan).^15^ To prepare the paper discs, a mixture of a 30-μL spore suspension and 10 μL Tris-HCl buffer was coated and dried on sterile paper discs. Subsequently, 10 μL of CuSO_4_ and 40 μL urine samples were added to the discs and incubated at 37°C.

### Patient groups

To determine the most suitable patient group for the paper-based kit, four groups of specimens were considered. (1) Urine samples from healthy individuals (no diabetes or CKD; Control), (2) urine samples from patients with type 2 diabetes without CKD; DIAB, (3) urine samples from patients with type 2 diabetes patients and CKD; DIAB-CKD, and (4) urine samples from patients with CKD. The paper-based biosensor was used to detect tyrosine in each sample.

### Analysis of tyrosine in urine samples by liquid chromatography-tandem mass spectrometry (LC-MS/MS)

For comparison, the tyrosine levels were also detected in the urine samples from the same patients with a conventional LC-MS/MS assay on the Agilent 1100 and SCIX API3000 systems with a C18 column. The mobile phase consisted of a distilled water/acetonitrile/methanol/trifluoroacetic acid/25% ammonium hydroxide solution (23/2/2/0.005/0.005) with a flow rate of 0.5 mL/min and a column temperature of 20°C. The MS conditions were as follows: capillary temperature, 270°C; sheath gas pressure, 0.80 MPa; aux gas pressure, 0.20 Mpa; source voltage, 4.5 kV.

## Results

### Tyrosine monitoring in urine

**Figure 1:**
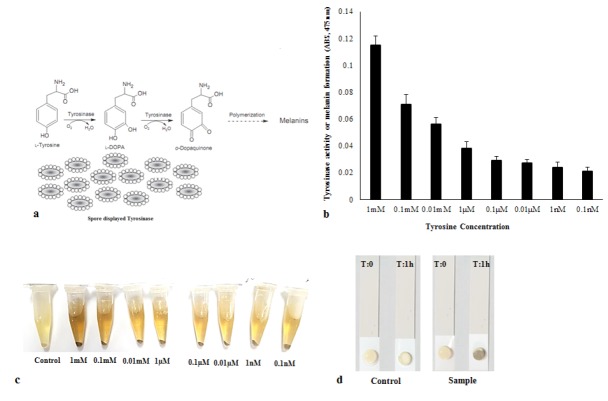
(a) The reaction of spore displayed tyrosinase with L-tyrosine as a substrate. (b, c) The reaction of dried spores with different concentrations of tyrosine (from 0.1nM to 1mM) in urine after 1 hour (the color spectrums and the optical density of different concentrations of tyrosine). (d) Paper based kit of spore displayed tyrosinase for detection of tyrosine in urine. Control: solution without tyrosine, Sample: solution with 0.1 µM tyrosine, T: time.

The reaction of spore-displayed tyrosinase with L-tyrosine as a substrate and melanin formation is shown in Figure 1a. In this reaction, L-tyrosine coverts to dopaquinone via tyrosinase to obtain the final melanin product. The color of the final reaction is directly related to the amount of melanin produced or the initial concentration of L-tyrosine, and varies accordingly along a gradient from dark yellow to black.

The spore-displayed tyrosinase system was used to detect different concentrations of tyrosine in urine according to a color spectrum from bright brownish-gray to dark brownish-gray, which was revealed within 1 hour. Melanin formation to different degrees could be clearly detected with various concentrations of tyrosine according to colorimetric methods, and the tyrosine activity was detected at 475 nm (Figure 1b, c). The spores were also coated and dried on sterile paper discs for the rapid detection of tyrosine in urine (Figure 1d). The results were obtained from five repeated tests.

### Tyrosine detection in patient groups using the spore-displayed tyrosinase paper-based kit

The amounts of tyrosine in the urine samples of patients from the three groups and the control group determined by LC-MS/MS are shown in Table 1. In comparison, the presence of different amounts of tyrosine in the urine samples was successfully detected by the spore-displayed tyrosinase paper-based kit. The color spectrum appeared on the spore-coated papers depending on the concentration of tyrosine in the samples of patient groups, indicating high sensitivity of the kit.

**Table 1:** The patient groups and tyrosine urinary excretion

**Group patients**	**No. of cases**	**Gender (male/female)**	**Age (y)**	**Tyrosine concentration in urine (nmol/mgCRT)**	**Paper based kit reaction**
Control	8	5/3	20-70	37- 52	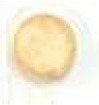
CKD	8	5/3	50-70	90- 115	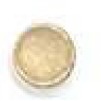
DIAB	9	6/3	50-70	123-130	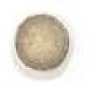
DIAB-CKD	11	7/5	50-70	145- 153	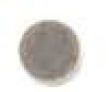

Group of patients: Control: Healthy persons, CKD: Chronic kidney disease, DIAB: Diabetics, DIAB: Diabetics- Chronic kidney disease. CRT: Creatinine. Interquartile ranges of the data are shown (*P* < 0.05).

## Discussion

Detection of tyrosine in biological samples is a useful marker of diabetes, CKD, and tyrosinemia.^5,16,17^ The risks of tyrosine excretion from the body are very serious, but these risks can be abated if tyrosinemia is detected earlier in newborns to allow for timely control of the disease or prevention of disease progression. Accordingly, the development of efficient screening tests for patients exhibiting tyrosinemia is very important.^18^ Chromatographic techniques are considered the most usable laboratory method to detect tyrosine in urine, with the ability to detect nanomolar concentrations.^7^ The Abcam company also introduced a colorimetric tyrosine assay kit based on the enzymatic oxidation of tyrosine, which could be detected by measuring the optical density value at 492 nm [http://www.abcam.com/tyrosine-assay-kit-colorimetric-ab185435.html]. We adapted this colorimetric assay from a solution-based assay to a paper discs-based assay, since spore-displayed tyrosinase can be active for a long period of time at room temperature.^15^ Thus, in our previous study, we successfully immobilized tyrosinase on the surface of *B. subtilis* spores using the genetic engineering spore surface display technique. We confirmed that the enzyme in the reaction with tyrosine as a substrate was stable for more than 15 days at room temperature. Furthermore, the spore-displayed tyrosinase could be used repeatedly up to six times.^15^ In the present study, we demonstrated a new application of this spore-displayed tyrosinase technique through creating a tyrosine detection kit.

## Conclusion

Herein, we developed a sensitive, inexpensive, rapid paper-based tyrosine assay kit using stable engineered spores that is usable at home or in laboratories for detection of tyrosinase in human and animal biological samples. This kit is not only widely applicable for personal health monitoring but is also an appropriate technology as routine a screening test for resource-limited countries. With our paper-based kit, the spore-displayed tyrosinase could monitor tyrosine in urine samples within 1 hour, detecting tyrosinase from only 0.01 nM up to 1 mM based on the formation of a color spectrum from bright brownish-gray to dark brownish-gray. Application of the kit to various patient groups further demonstrated the ability to screen various diseases according to different concentrations of tyrosine based on the color spectrum.

## Ethical Issues

The study protocol was approved by research committee of University of Isfahan in 28/09/2017 (Reference number: 96/25672). A written informed consent document also was obtained from all participants.

## Conflict of Interest

The authors declare that they have no conflict of interest.

## Acknowledgments

The authors would like to thank the University of Isfahan for financial support to the MSc student. This work is a collaborative project between University of Isfahan, Iran and Seoul National University, Korea for the MSc thesis. This research was partly supported by a grant (NRF-2017R1D1A1B05035726) from the Basic Science Research Program through the National Research Foundation of Korea (NRF) funded by the Ministry of Education, Republic of Korea.
